# BARRIERS AND SOCIETAL FACTORS SURROUNDING LONG-STAY RESIDENT DISCHARGE TO THE COMMUNITY IN LOUISIANA

**DOI:** 10.1093/geroni/igad104.3200

**Published:** 2023-12-21

**Authors:** Valerie Williams

**Affiliations:** AHCA/NCAL, Washington, District of Columbia, United States

## Abstract

The focus of this study is to determine the resident characteristics and societal factors that prevent long-stay residents (LSR) from discharging to the community based on the LSR in Louisiana (LA) compared to the United States (US). This study will also explore solutions that can assist LSR in successfully discharging to the community. Discharge to the community from a skilled nursing facility is influenced by functional status, health of the resident, and the availability of community-based services. About 20% of LSR (>100 days) discharge to the community from skilled nursing facilities (SNF), while that number is more than doubled for short-stay residents. Current data provides little information about the odds of a LSR returning to the community. CMS data shows that LSR in LA have higher rates of Activities of Daily Living Decline (ADL) (LA-18.83%/US-14.68%), Antipsychotic Use (LA-16.9%/US-14.58%), and Pressure Ulcers (LA-9.4%/US-2.4%) than the national average. LSR exhibiting these characteristics are less likely to be discharged to the community. Additional characteristics of this population include high levels of comorbid chronic conditions that are difficult to manage outside of the facility, and having Medicaid (LA-71.5%/US-61.1%) as their payer status, a known barrier to community discharge. States should increase the availability of home health and community-based services that cater to the Medicaid population. Assisted living facilities are a great alternative to living “at home”. Additional research should be conducted to examine affordable assisted living options in more states for long-stay Medicaid residents who desire to live independently in the community.

